# Action-Related Thoughts and Beliefs Regulate the Effect of Age Stereotypes on Aging Preparation

**DOI:** 10.1093/geroni/igab046.729

**Published:** 2021-12-17

**Authors:** Yaeji Kim-Knauss, Frieder Lang, Helene Fung, Dwight Tse

**Affiliations:** 1 Institute of Psychogerntology, University of Erlangen-Nuremberg, Nuremberg, Bayern, Germany; 2 Institute of psychogerontology, University of Erlangen-Nuremberg, Nuremberg, Bayern, Germany; 3 The Chinese University of Hong Kong, Shatin, N.T., Hong Kong, Hong Kong; 4 School of Psychological Sciences and Health, University of Strathclyde, Glasgow, Scotland, United Kingdom

## Abstract

Thinking about old age stereotypically affects one’s engagement in age-related behaviors and developmental regulation. We hypothesized that positive or negative aging stereotype (AS) would be associated with more or less aging preparation, while action-related thoughts and beliefs might exert influence thereon. We used the AAF online-study dataset consisting of 591 German, 348 Chinese, and 139 American adults (aged 18−93 and 55% female). Using a count measure of 15-preparatory-activities, we first explored the role of AS measured by a bipolar scale and how perceived utility and risk of aging preparation differentiate this association. Findings revealed that perceiving more utility buffered the impact of negative AS, which suggests that one’s action-related thoughts are more proximal and self-relevant predictor of aging preparation. Besides, Chinese and Americans were more susceptible to the presence of AS than Germans, implying that cultural background or societal conditions might also shape one’s belief system and thereby regulate behaviors.

